# NCAM180 Regulates Ric8A Membrane Localization and Potentiates β-Adrenergic Response

**DOI:** 10.1371/journal.pone.0032216

**Published:** 2012-02-22

**Authors:** Marie-Claude Amoureux, Stéphane Nicolas, Geneviève Rougon

**Affiliations:** 1 Institut de Biologie du Développement de Marseille-Luminy, Aix-Marseille Université CNRS 6216, Marseille, France; 2 Eurobio/Abcys, Les Ulis, France; INSERM U894, France

## Abstract

Cooperation between receptors allows integrated intracellular signaling leading to appropriate physiological responses. The Neural Cell Adhesion Molecule (NCAM) has three main isoforms of 120, 140 and 180 kDa, with adhesive and signaling properties, but their respective functions remains to be fully identified. Here we show that the human NCAM180 intracellular domain is a novel interactor of the human guanosine exchange factor (GEF) Ric8A using the yeast two hybrid system and immunoprecipitation. Furthermore, NCAM, Ric8A and G_αs_ form a tripartite complex. Colocalization experiments by confocal microscopy revealed that human NCAM180 specifically induces the recruitment of Ric8A to the membrane. In addition, using an *in vitro* recombinant system, and *in vivo* by comparing NCAM knock-out mouse brain to NCAM heterozygous and wild type brains, we show that NCAM expression dose dependently regulates Ric8A redistribution in detergent resistent membrane microdomains (DRM). Previous studies have demonstrated essential roles for Ric8 in G_α_ protein activity at G protein coupled receptors (GPCR), during neurotransmitter release and for asymmetric cell division. We observed that inhibition of Ric8A by siRNA or its overexpression, decreases or increases respectively, cAMP production following β-adrenergic receptor stimulation. Furthermore, in human HEK293T recombinant cells, NCAM180 potentiates the G_αs_ coupled β-adrenergic receptor response, in a Ric8A dependent manner, whereas NCAM120 or NCAM140 do not. Finally, in mouse hippocampal neurons expressing endogenously NCAM, NCAM is required for the agonist isoproterenol to induce cAMP production, and this requirement depends on Ric8A. These data illustrate a functional crosstalk between a GPCR and an IgCAM in the nervous system.

## Introduction

NCAM comprises three major alternatively spliced isoforms [1]. While their extracellular domains are identical, NCAM180 has a 267 amino acid insert in the intracellular domain compared to NCAM140, and NCAM120 is glycophosphatidylinositol (GPI)-anchored at the membrane. However, the specialized functions of each NCAM isoform and their respective interacting partners remain incompletely elucidated. NCAM mediates a variety of cell–cell interactions important for neural development, synapse formation and synaptic plasticity. Some of these effects may be attributable to NCAM acting as a cell adhesion molecule whereas others result from a complex network of intracellular signaling cascades [2]–[4]. As the signal transduction of extracellular cues depends on multimolecular receptor complexes and modulators of intracellular effectors, it is anticipated that interactions and cooperative mechanisms exist between NCAM isoforms and proteins with previously unsuspected related functions.

Seven transmembrane G protein coupled receptors (GPCR), coupled to G_αβγ_ proteins, have been characterized extensively and generate responses to a wide range of stimuli in physiological or diseased conditions. The equilibrium between the inactive and active G_α_ is modulated by GTPase activating proteins, non-receptor GEFs, and Guanine dissociation inhibitors [5]. The Ric8 family has two known members, Ric8B and the evolutionary conserved Ric8A. In mammals, Ric8A acts broadly on monomeric G_α_ proteins [6]. In Drosophila [7], [8] as in *Caenorhabditis elegans (C.elegans)* [9] or mammalian cells [10], Ric8A is critical to asymetric cell divisions. Interestingly, *C. elegans* Ric8A knock out is defective in neurotransmitter secretion and in synaptic vesicle priming [11]. The resulting paralysis phenotype can be rescued by genes of the G_αq_ and G_αs_ pathways [12].

Overlapping functions of Ric8A, NCAM180 and β-adrenergic receptors have been described in neurotransmitter release [11], [13]–[15], or mouse behavioral phenotypes [16]–[19]. Here, we demonstrate that these 3 molecules are functionally linked, hence tying a cell adhesion molecule to GPCR signaling in the nervous system.

## Materials and Methods

### Ethics

Animals were treated according to guidelines of the French ethical committee who approved the study (approval ID: E13-055-21).

### Yeast two-hybrid screening

Yeast two-hybrid screening was performed using the ProQuest™ system (Invitrogen). The complete coding region of human NCAM180 and NCAM140 intracellular domains (NCAM_180cyto_ and NCAM_140 cyto_) were obtained by PCR from the pRC/CMV plasmids containing each full lenght NCAM isoforms [20], and subcloned into a pDONR221 plasmid using Gateway® Technology (Invitrogen). Recombination with pDEST32 plasmid generated the yeast bait constructs DNA-Binding (DB)-NCAM_180cyto_ and DB-NCAM_140cyto_. The prey corresponded to the human adult brain ProQuest cDNA library fused to the GAL4 activation domain in the pEXP-AD22 plasmid. Yeast MaV203 competent cells were co-transformed by the library and DB-NCAM_180cyto_ or DB-NCAM_140cyto_. Positive clones from screening were sequenced (Eurofins MWG Operon) and identified using BLAST analysis.

### Antibodies, immunoprecipitation and GST-pull down

Purified full lenght Ric8A-GST was used to produce a rat anti-Ric8A antibody (Eurogentec). A goat polyclonal NCAM antibody was produced (Eurogentec) using immunopurified mouse of either sex brain NCAM. For immunopurification, H28 anti-NCAM monoclonal antibody [21] was covalently coupled to Aminolink beads (Pierce) (10 mg antibody/ml resin). Purification of IgG fractions from anti-Ric8A and anti-NCAM sera was performed using protein G beads (Pierce). The specificity of the anti-NCAM antibody was assessed on NCAM knock out brain extract for which no signal was detected and HEK293T cells transfected with each NCAM isoform (not shown). The antibody was able to detect all three NCAM isoforms.

For immunoprecipitation, anti-NCAM was immobilized on aldehyde activated AminoLink beads. Control beads were AminoLink beads quenched with 1 M TrisHCl. Anti-NCAM beads were incubated with female mouse brain or HEK293T cells extracts in PBS+0.1% Nonidet P-40 (NP-40) (overnight, 4°C), washed 3 times with PBS, and bound proteins eluted with β-mercaptoethanol containing Laemli buffer.

For GST-pull down, female mouse brain extract was cleared with GSH beads (1 h, 4°C). The cleared lysate was incubated with recombinant Ric8A-GST (3 h, RT). GSH beads were added to this mixture (1 h, 4°C). The beads were washed, bound proteins eluted as above, and NCAM detected by Western blot. Control beads consisted of GSH beads incubated with purified GST and cleared lysate. Rat anti-Ric8A (1∶4000), anti-NCAM (1∶3000), anti-caveolin3 (1∶2000; Abcys), and rabbit anti-G_αs_ antibody (K20, 1∶500; Ozyme) were used.

### Immunofluorescence

Neurons transfected with Ric8A-GFP, or HEK293T cells co-transfected with Ric8A-GFP and NCAM120, NCAM140 or NCAM180 were fixed after 24 h with 4% parafomaldehyde and stained with goat polyclonal anti-NCAM (1∶1000, 2 h, RT) followed by Alexa555-anti-goat antibody (1∶500, 1 h, RT, Jackson Laboratory). Confocal images were obtained using a LSM510 Meta UV microscope (Zeiss).

### Inhibition of Ric8A by RNA interference

Inhibition of endogenous Ric8A in HEK293T cells and neurons was performed using human and mouse Ric8A pools of siRNA (OnTarget Smart pools, Thermo Fisher Scientific Dharmacon) designed to have no off-target effects [22]. The target sequences are GGGGAGAUGCUCGGAACA, AGAACUUUCCAUACGAGUA, CAGGAUGCCAUGUGCGAGA and CAGAGGAGUUCCACGGCCA for human Ric8A and GAGAGUAGCUGCCGAGUUC, GUGUGGGACUGUACCGCAA, GUACACAGGCUACGGGAAU and CAGAGGAGUUCCACGGCCA for mouse Ric8A.

The efficiency of inhibition was verified in COS-7 cells transfected with human Ric8A-GFP, and in HEK293T cells transfected or not with NCAM180 (Suppl Fig. S1). SiGLO RNA-induced silencing complex-free control siRNA, a fluorescent siRNA without RNA target (Thermo Fisher Scientific Dharmacon) was used as control siRNA in all experiments (siCtrl). The sequences of Ric8A siRNA had no homology with Ric-8B, therefore could not inhibit Ric8B transcription. Transfections of siRNA (0.1 nmol per 10^6^ cells) were performed using the Amaxa electroporation system (kit V for HEK293T cells and mouse neuron nucleofector kit for neurons) as indicated by the manufacturer (Lonza).

### Cell culture, cloning and transfections

All cell lines were from American Tissue Culture Collection. COS-7 and HEK293T cells were grown in DMEM with 10% FCS, penicillin (50 Units/ml) and streptomycin (100 µg/ml) (Invitrogen). Mouse embryonic cortical and hippocampal neurons were prepared as described and plated in Neurobasal medium (NB) with B27 supplement [23]. Human Ric8A was cloned in pDEST48-GFP, pDEST15-GST and pcDNA3-V5 tagged plasmid using the Gateway system procedures. The 3 full length NCAM isoforms were in pRC/CMV [20]. Pmax-GFP (Lonza) was used as control plasmid. Ric8A-GST was produced after IPTG induction of DH-5α bacteria and purified on GSH-Sepharose fast flow beads (GE Healthcare). Transfections of plasmids (2 µg for 10^6^ cells) and siRNA (0.1 nmol per 10^6^ cells) were performed using the Amaxa electroporation system (Lonza). For PSA-NCAM-Fc production, TE671 cells, which express polysialyltransferases [20], were transiently transfected using lipofectamine (Invitrogen) according to the manufacturer's instruction in OptiMEM medium (Invitrogen). pIG1 expression plasmid encoding NCAM-Fc was kindly provided by Dr Simmons (Cell Adhesion Laboratory) and previously used in our laboratory [24]. Culture supernatants were harvested 60 h after transfection, and Fc-bearing secreted PSA-NCAM was affinity-purified on a protein-A column (Pierce). The affinity-isolated proteins were analysed under reducing conditions by SDS-PAGE. The resulting purified solution was then concentrated and buffer exchanged in PBS using Centricons (Millipore), and adjusted to a final concentration of 10 µg/ml.

### Preparation of DSM and DRM fractions

Cells or tissue (brain from male mice) lysis was performed by sonication of a given volume (V) of ice-cold 10 mM Tris HCl pH 7.4 containing Complete™ protease inhibitors (PI; Roche). The nuclear and mitochondrial fractions, and debris were removed by centrifugation (10000 g, 4°C, 15 min). The supernatant (corresponding to the cytosolic fraction) was spun down (100000 g, 1 h, 4°C). The pellet, corresponding to the membrane fraction, was resuspended in the initial volume V of PBS+PI+1% NP40, and sonicated. This extract was spun down (100000 g, 1 h, 4°C). The supernatant corresponded to DSM and the pellet to DRM. The later was sonicated in the same volume V of PBS+PI+1% NP40. The quality of preparation of DSM and DRM was monitored by the presence of caveolin-3 in DRM and DSM, a criterion previously used [25], [26].

### β-adrenergic receptor stimulation, Phosphorylated-ERK (P-ERK) and cAMP measurements

HEK293T cells or hippocampal neurons were transfected and plated in 24-well plates (180000 HEK293T cells/well or 500000 neurons/well). The next day, HEK293T cells were placed in serum free DMEM for 24 h. Neurons or HEK293T cells were washed with HBSS containing 1 mM isobutylmethylxanthine, and treated with isoproterenol for 20 min. Medium was removed and cellular cAMP measured using the kit Parameter™ (R&D Systems).

P-ERK was quantified by Western blot from hippocampal neurons extracts (PBS+1%NP-40) after 1.5 h of culture in NB medium, followed by 5 min 10 µM isoproterenol treatment, using anti-actin (AC-40, 1∶1000, Sigma) and anti-phopho-ERK1/2 (20G11, 1∶1000; Ozyme).

### Neurite outgrowth measurement

For neurite outgrowth experiments, cortical neurons were prepared from embryonic stage E15–16 mouse cortex as previously described in details for hippocampal neurons [27]. Briefly, cortices of 6 embryos were dissected out and digested with Trypsin (Invitrogen). The digestion was stopped by adding NB medium supplemented with 10% FCS. After centrifugation, the tissue was mechanically dissociated and cells counted. Neurons were cultured in NB medium supplemented with 0.5 mM Glutamax, B27 (Invitrogen), and 25 µM L-glutamine (Sigma). 1.5×10^3^ cells/ml were plated in 96-well black plates (Greiner). Coating conditions were either 10 µg/ml poly-D-Lysine (Sigma) alone or in combination with PSA-NCAM-Fc protein (10 µg/ml) coated overnight after poly-D-lysine. Such cultures contained more than 95% neurons. After 3 days in culture, live cells were stained with 0.5 µg/ml green calceine-acetoxymethyl ester (Molecular Probes) and images of the entire wells captured using a flash cytometer (Trophos) (8 wells per condition). Neurite outgrowth was measured using the Metamorph software (Molecular Devices).

### Statistical analysis

Statistical significance was achieved for p<0.05 (*), p<0.01 (**) and p<0.001 (***). One-way ANOVA was used when more than 2 groups were compared, followed by the Bonferroni post-hoc test. The t-test was used when 2 groups were compared.

## Results

### NCAM180 interacts with Ric8A

In an attempt to identify interactors of the intracellular domains of NCAM180 (NCAM_180cyto_) and NCAM140 (NCAM_140cyto_), a yeast two-hydrid screen was performed using NCAM_180cyto_ or NCAM_140cyto_ as baits and a human adult cDNA library as prey. We focussed our study on the interactor Ric8A, as we found it interacted with NCAM_180cyto_, but not with NCAM_140cyto_. One of the Ric8A clones present in the library and positive for NCAM_180cyto_, but not with NCAM_140cyto_ covered 90% of the Ric8A entire sequence, suggesting the specificity of the interaction of Ric8A with NCAM180.

**Figure 1:**
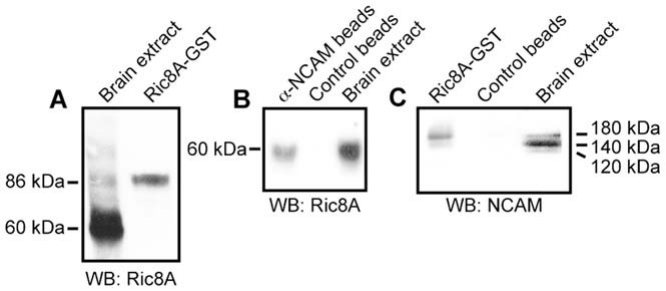
Biochemical analysis of Ric8A-NCAM interaction. A- Characterization of the developed rat anti-Ric8A antibody by Western blot reveals a protein of 60 kDa, which is the expected molecular weight for Ric8A, from a brain extract. The GST coupled to Ric8A leads to a molecular weight of 86 kDa. B- Ric8A was immunoprecipitated from mouse brain extract with Aminolink beads coupled to a polyclonal anti-NCAM antibody but not with control beads. C- NCAM180 was pulled down from a mouse brain extract containing the 120, 140 and 180 kDa NCAM isoforms by Ric8A-GST but not by control beads.

To further demonstrate an interaction between NCAM and Ric8A, we performed co-immunoprecipitation experiments from mouse brain extract. An antibody against the human recombinant protein Ric8A-GST was generated and recognized Ric8A-GST and a protein of 60 kDa corresponding to Ric8A in mouse brain extract (Fig. 1A). Ric8A was detected in immunocomplexes after immunoprecipitation from a brain extract with beads coupled to anti-NCAM, but not with control beads (Fig. 1B). Moreover, Ric8A-GST, but not GST control beads, pulled down NCAM180 from brain extract (Fig. 1C). Altogether, these data indicate that the intracellular domain of NCAM180 interacts with Ric8A.

### NCAM180 allows recruitment of Ric8A at the cell membrane

**Figure 2:**
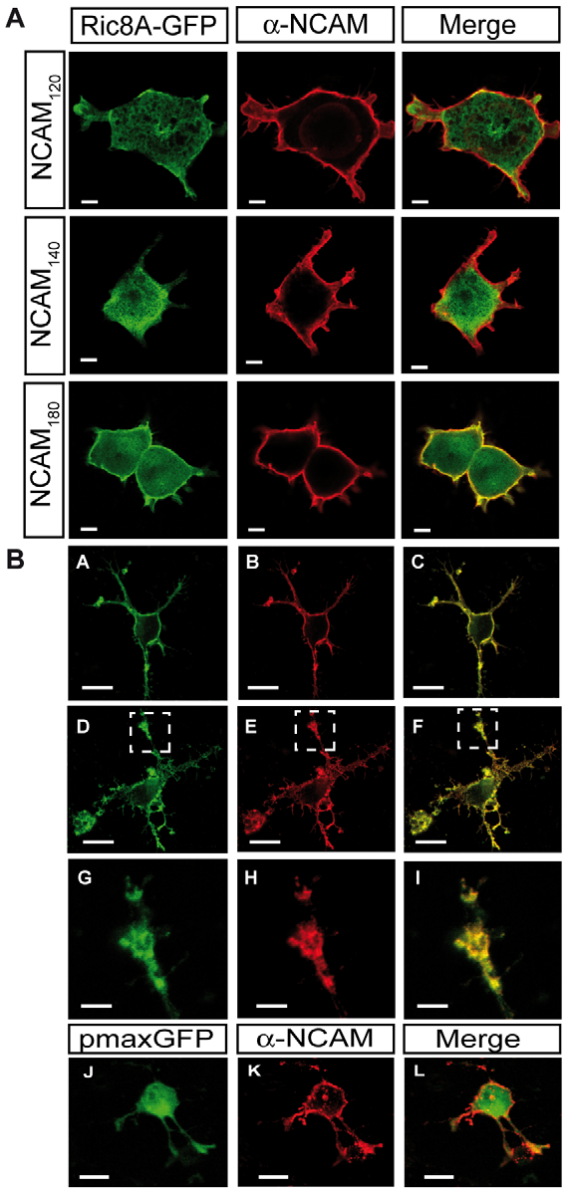
NCAM180 induces Ric8A-GFP recruitement at the membrane where NCAM and Ric8A colocalize. A- Ric8A-GFP co-transfected with NCAM120, NCAM140 or NCAM180 in HEK293T cells co-localizes specifically with the NCAM180 isoform. Scale bar: 10 µm. B- Ric8A-GFP or PmaxGFP plasmids were transfected in cortical neurons for 24 h. Images were taken focussing at the membrane plane with a 20× objective. NCAM staining is clearly associated to the cell membrane (B, E, K). Ric8A-GFP is strongly localized at the membrane at the level of the cell body and processes (A, D). PmaxGFP control plasmid is mostly found in the cytoplasm (J). Ric8A-GFP, but not PmaxGFP colocalizes with NCAM at the membrane (Ric8A/NCAM merge: C, F versus Pmax/NCAM merge: L). (G–I): higher magnification pictures (dotted line box D–F) using a 63× oil immersion objective indicate a strong co-localization of Ric8A-GFP with NCAM in the growth cone (I). Scale bars: 10 µm (A–F, J–O); 1 µm (G–I).

**Figure 3:**
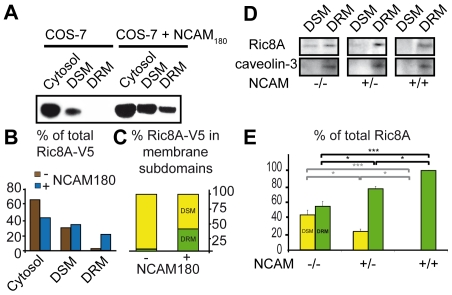
NCAM affects Ric8A relative distribution in DSM and DRM fractions *in vitro* and *in vivo*. A- Western blot of overexpressed Ric8A-V5 in cytosol, DSM and DRM. B, C- Quantitative analysis of Western blots in A. D- Western blot of Ric8A in DSM and DRM from brain extracts prepared from NCAM null (−/−), heterozygous (+/−) and wild type (+/+) mice. Caveolin-3, a marker for enrichment of lipid rich domains was detected only in DRM. E- Quantitative analysis of Western blots presented in D (n = 3 per genotype). Data are expressed as mean +/− SEM; *p<0,05, **p<0,01, ***p<0,001. One-way ANOVA for more than 2 groups was performed. The effect of the single factor ‘genotype’ was then analysed by the Bonferroni post-hoc test.

To further investigate the interaction of NCAM with Ric8A, we transfected HEK293T cells with Ric8A-GFP and the three different NCAM isoforms. We showed by confocal imaging that when NCAM180 was overexpressed in HEK293T cells (Fig. 2A), Ric8A-GFP was strongly colocalized to NCAM staining at the membrane. Similarly, in neurons transfected with Ric8A-GFP, there was a strong colocalization between Ric8A-GFP and endogenous NCAM at the membrane and in growth cones (Fig. 2B). Conversely, when NCAM120 or NCAM140 was overexpressed in HEK293T cells, Ric8A-GFP was mostly found in the cytoplasm, and very slightly colocalized with NCAM120, or NCAM140 at the membrane of HEK293T cells, compared to NCAM180 (Fig. 2A). Pearson correlation coefficients for colocalization in HEK293T cells were derived from the LSM Zeiss software colocalization scatterplot coordinate system by selecting the region of the entire membrane area, and were 0.22, 0.38 and 0.66 for NCAM120, NCAM140 and NCAM180, respectively. Recruitment of Ric8A at the membrane by NCAM180 was confirmed by a biochemical approach in COS-7 cells co-transfected with V5-tagged Ric8A and NCAM180 (Fig. 3). We performed subcellular fractionation of the cytosol, detergent soluble (DSM) and resistent (DRM) membrane subdomains (Fig. 3A). Total Ric8A-V5 was used as its own internal control in each cell line. When NCAM180 was expressed, the percentage of Ric8A-V5 present in the cytosol decreased from 68 to 43% and increased from 32 to 57% in the total membrane fraction (DSM+DRM). Therefore, in contrast to NCAM120 and NCAM140, NCAM180 colocalized with Ric8A and specifically induced trafficking of Ric8A from the cytoplasm to the membrane.

### NCAM controls the presence of Ric8A in detergent resistant membrane domains

We noticed that Ric8A distribution in the subcellular compartements DSM versus DRM was affected by NCAM180 overexpression in COS-7 cells (Fig. 3B, C). In COS-7 cells overexpressing NCAM180, 40% of membranous Ric8A was shifted to DRM (Fig. 3C). We used brain extracts from genetically modified NCAM knockout mice to analyze how NCAM influenced Ric8A distribution between DSM and DRM (Fig. 3D). Whether NCAM was expressed or not, Ric8A was never detected in the cytosol (not shown), suggesting that in the brain, endogenous Ric8A is predominantly present at the membrane. We compared endogenous Ric8A expression in NCAM deficient (−/−), heterozygous (+/−) and wild type (+/+) mouse brains. Total Ric8A amount was not different between genotypes (not shown). The presence of the marker caveolin-3 for the DRM fraction was not affected by the genotype (Fig. 3D). In brain extracts from NCAM wild type (+/+) mice, Ric8A was exclusively found in the DRM fraction, whereas loss of NCAM (−/−) induced a redistribution of Ric8A, which was approximately even between the DRM (56%) and DSM fractions (44%) (Fig. 3D, E). These data indicate that in adult wild type mouse brain, NCAM maintains 44% of Ric8A in DRM. Moreover, NCAM is a dose-dependent determinant of this process since a half dose of NCAM (+/−) resulted in half of this amount (23%) remaining localized in DSM (Fig. 3D, E).

To further elucidate whether the isoform NCAM180 had a specific and active direct role in the translocation of Ric8A from DSM to DRM, we used HEK293T cells expressing recombinant NCAM140 or NCAM180. Endogenous Ric8A was exclusively found in the membrane fraction as in mouse brain. Similar proportions of Ric8A were present in DRM and DSM fractions in control or NCAM140 transfected HEK293T cells. By contrast, NCAM180 expression led to a strong prevalence of Ric8A in DRM. Ric8A was 2.7+/−0.4 (mean +/− SD) times more abundant in DRM than in DSM in NCAM180 expressing cells than in control cells (p<0.01) or NCAM140 expressing cells (p<0.05; 3 independent cultures; ANOVA, Bonferroni post-hoc test) expression. The percentage of Ric8A translocated to DRM by NCAM180 was 46% of total Ric8A. Altogether, these data indicate that NCAM180 specifically induces a relocalization of endogenous Ric8A from DSM to DRM domains.

### Ric8A is not involved in PSA-NCAM induced neurite outgrowth

**Figure 4:**
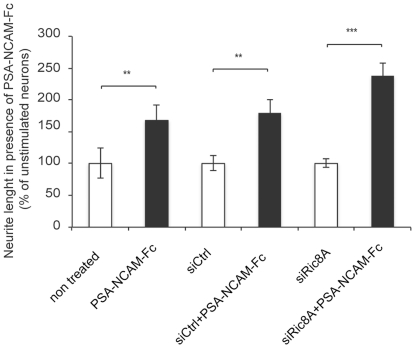
Ric8A is not involved in NCAM induced cortical neurite outgrowth. Embryonic mice cortical neurons were plated on poly-D-lysine alone or in combination with PSA-NCAM-Fc. Non transfected neurons, or neurons transfected with siCtrl or siRic8A were either not treated or PSA-NCAM-Fc treated. After 3 days in culture, neurons were stained with calcein as described in Materials and Methods, and neurite length was quantified using the Metamorph software. Data are from a representative experiment (out of 5 performed) and are expressed as mean +/− SEM. One-way ANOVA for more than 2 groups was performed. The effect of PSA-NCAM-Fc treatment, was then analysed by with the Bonferroni post-hoc test.

The involvement of G_αi_/G_αo_ coupled receptor channels in trans homophilic NCAM stimulated neurite outgrowth has been previously reported [28], [29]. We set up to investigate whether Ric8A could also be involved. To this end we treated cortical neurons with soluble PSA-NCAM-Fc, which very significantly increased neurite outgrowth. Treatment with either control siRNA or siRic8A did not affect the stimulation of growth (Fig. 4). Moreover, no effect of Ric8A was found on the number of processes or their branching (not shown).

### NCAM-Ric8A interaction modulates β-adrenergic response in HEK293T cells

**Figure 5:**
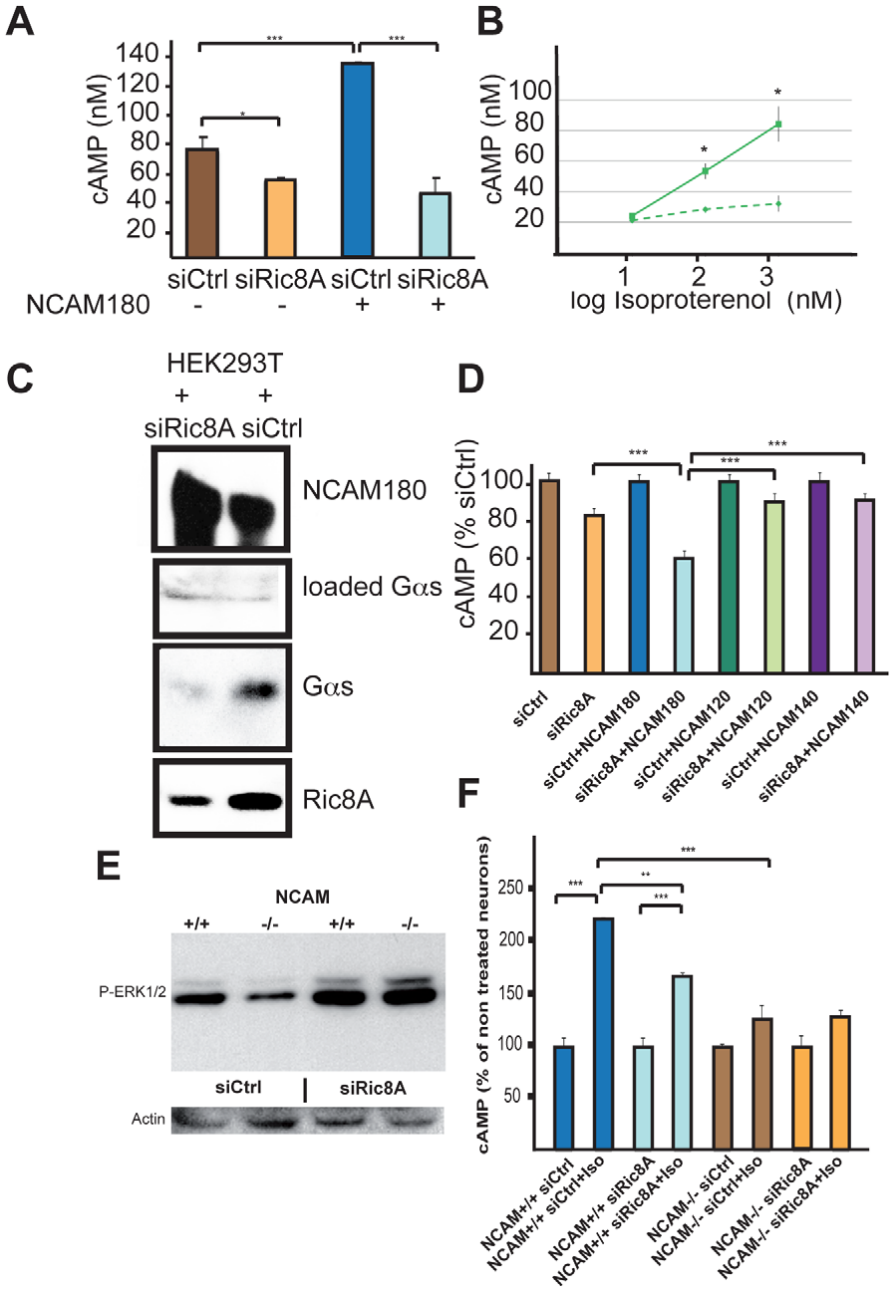
NCAM180 regulates β-adrenergic response via Ric8A in HEK293T cells and neurons. A- Representative experiment (out of 3 performed) of isoproterenol (10 µM) induced cAMP production in HEK293T cells transfected or not with NCAM180, in the presence (siCtrl) or absence (siRic8A) of Ric8A. Data are expressed as mean +/− SEM of 3 independent wells and analyzed by ANOVA and Bonferroni post-hoc test. B- Increase of cAMP production by Ric8A overexpression in HEK293T cells transfected for 48 h with Ric8A-GFP (▪) or PmaxGFP (⧫). Data are expressed as mean +/− SEM of 3 independent wells and analyzed by the t-test. C- HEK293T cells were transfected with NCAM180, and control or Ric8A siRNA for 24 h. Anti-NCAM beads were used for immunoprecipitation of cell extracts, A Western blot with anti-NCAM (first box) indicates that NCAM180 has been immunoprecipitated. A Western blot was also performed with anti-G_αs_ on each extract before immunoprecipitation to check the amount of loaded G_αs_ (second box). Anti-G_αs_ (third box) and anti-Ric8A (fourth box) were used on NCAM180 immunoprecipitated extracts and show that Ric8A is significantly decreased by siRic8A compared to siCtrl, and that G_αs_ is immunoprecipitated when Ric8A is present (siCtrl).. D- Amount of cAMP generated by isoproterenol (10 µM) that led to maximal efficacy in HEK293T, or HEK293T cells transfected with the different NCAM isoforms in the presence (siCtrl) or absence (siRic8A) of Ric8A. Data are expressed as % of siCtrl transfected cells and as mean +/− SEM of at least 3 independent experiments. Data were analyzed by ANOVA for more than 2 groups and Bonferroni post-hoc test. E- P-ERK1/2 induction by isoproterenol (10 µM) in hippocampal neurons from NCAM +/+ and −/− E15.5 embryos, in the presence (siCtrl) or absence (siRic8A) of Ric8A. Actin was used as constitutive protein. F- Amount of cAMP produced by NCAM^+/+^ and NCAM^−/−^ non treated hippocampal neurons or after treatment with 1 µM isoproterenol in the presence (siCtrl) or absence (siRic8A) of Ric8A. Data are expressed as percentage of non-treated neurons (mean +/− SEM of 3 independent cultures). One-way ANOVA for more than 2 groups was performed. The effect of the single factor ‘isoproterenol treament’ was then analysed by the Bonferroni post-hoc test.

We further studied the functional significance of the NCAM-Ric8A interaction by postulating that NCAM could affect GPCR signaling in which Ric8A is known to play a GEF regulatory role. Furthermore, as G_αs_ localization in DRM is increased after β-adrenergic agonist stimulation [30], we postulated that the observed redistribution of Ric8A in DRM could also affect β-adrenergic signaling. We used the agonist isoproterenol to target the G_αs_ coupled β-adrenergic receptors present in HEK293T cells. We compared the amount of cAMP produced by 10 µM isoproterenol in several conditions: the absence or presence of NCAM180, the inhibition of human Ric8A by siRNA, and the overexpression of Ric8A. Our analyses first revealed that siRic8A, which induced an 80% decrease of Ric8A expression (Suppl Fig. S1), inhibited cAMP production in the absence or presence of NCAM180 (Fig. 5A). Second, when Ric8A was overexpressed in HEK293T cells, cAMP production was dramatically increased (Fig. 5B). This shows that Ric8A allows the potentiation of β-adrenergic response. Unstimulated HEK293T cells had similar levels of cAMP in all conditions (siCtrl: 0.87+/−0.07 nM; siRic8: 0.40+/−0.05 nM; siCtrl+NCAM180: 0.85+/−0.26 nM; siRic8A+NCAM180: 0.51+/−0.12 nM in a representative experiment out of 3 performed), indicating that NCAM does not affect basal cAMP homeostasis and that its effect is dependent on receptor activation. Third, G_αs_ was immunoprecipitated by NCAM, along with Ric8A, in a Ric8A-dependent manner (Fig. 5C). Therefore NCAM does not directly interact with G_αs_, as Ric8A is required for G_αs_ pull down, strongly suggesting that NCAM and Ric8A are part of a complex with G_αs_. Fourth, NCAM180 enhanced β-adrenergic response by 1.75 fold, in an entirely Ric8A dependent manner (Fig. 5A). Finally, inhibition of Ric8A decreased β-adrenergic response more efficiently in the presence of NCAM180 than in its absence, whereas this observation was not made for the two other NCAM isoforms (Fig. 5D), supporting the specific functional link between Ric8A and NCAM180.

### NCAM-Ric8A interaction modulates β-adrenergic response in hippocampal neurons

To investigate the role of Ric8A in cells that endogenously express NCAM180, we compared isoproterenol induced β-adrenergic response in hippocampal neurons by measuring P-ERK (Fig. 5E) and cAMP production in wild type and NCAM deficient neurons (Fig. 5F). P-ERK induction by isoproterenol normalized for actin from NCAM^−/−^ was 32% that of NCAM^+/+^ neurons. This difference was abolished when Ric8A was inhibited (Fig. 5E). Moreover, whereas 1 µM isoproterenol increased cAMP by 2.25-fold in NCAM^+/+^ neurons, this induction was significantly lower (1.68-fold; p<0.01) when Ric8A was inhibited by siRNA (Fig. 5F) indicating that Ric8A also potentiates β-adrenergic response in neurons. Basal cAMP levels were however identical in non-treated NCAM^+/+^ and NCAM^−/−^ neurons (NCAM^+/+^/siCtrl: 4.48+/−0.74 nM; NCAM^+/+^/siRic8A: 5.83+/−0.95 nM; NCAM^−/−^/siCtrl: 3.01+/−0.18 nM; NCAM^−/−^/siRic8A: 4.06+/−0.82 nM in a representative experiment out of 3 performed). In addition, 1 µM (Fig. 5F) or 10 µM (not shown) isoproterenol was unable to induce a significant production of cAMP in NCAM^−/−^ neurons in the presence or absence of Ric8A. These results indicate that in neurons, NCAM is required for a β-adrenergic response, and this requirement is at least partly dependent on Ric8A.

## Discussion

In this study, a novel interactor of NCAM, Ric8A was identified. We report an interaction between NCAM180 and Ric8A. In contrast to NCAM120 and NCAM140, NCAM180 colocalized with Ric8A and specifically induced trafficking of Ric8A from the cytoplasm to the membrane, and particularly to DRM. Earlier observations reported a specific interaction of NCAM_180cyto_ with β spectrin [31]. However, a direct interaction was later shown to occur also with NCAM140 [32]. The two-hybrid data, together with biochemical and immunocolocalization analysis suggest that Ric8A may interact preferentially with the cytoplasmic domain of NCAM180.

Functionnally, our results showed that in HEK293T cells, Ric8A potentiates a β-adrenergic response, that NCAM180, Ric8A and G_αs_ form a molecular complex, and that NCAM180 enhances β-adrenergic response only in the presence of Ric8A, without modulating basal cAMP homeostasis. Moreover, constitutive NCAM expression in hippocampal neurons is required to induce cAMP production upon agonist stimulation, and a NCAM-Ric8A interaction is potentially involved in β-adrenergic response in the brain.

These results point to the role of constitutively expressed NCAM and Ric8A in neurons and reveal an unsuspected NCAM180 specific function in modulating a crucial intracellular step in G_α_-dependent signaling pathways. Ric-8 has previously been shown to be a GEF for G_αs_ in Xenopus [33], and mammalian Ric8A to interact with G_αs_ [34]. However, Tall et al [6] did not show binding of G_αs_ to rat Ric8A by yeast two-hybrid screen. This discrepancy could be explained by the fact that interaction in yeast does not take into account posttranslational modifications predicted for Ric8A protein [6]. Hein et al [35] showed that G_αs_ activation is the rate-limiting step of GPCR response. Therefore, NCAM180, by interacting with the G_αs_ signal amplifier Ric8A, and forming a tripartite complex with G_αs_ and Ric8A, could increase the rate of activation of G_αs_ and/or increase the availability of these effectors to the appropriate subcellular compartments for signaling. DRM enriched in lipid rafts play a crucial role in sorting mechanisms of receptors and effectors involved in signaling [36]. Specifically, G_αs_ localization in DRM is increased after β-adrenergic agonist stimulation [30]. Moreover, Ric8A controls G_α_ localization required during asymetric division [7]–[9]. We showed that NCAM180 expression was specifically associated with the anchoring of Ric8A at the membrane and its enrichment in the DRM subdomains in cultured cells and *in vivo*, in mouse brain. In brain, the relative presence of Ric8A in DSM and DRM subdomains was directly proportional to the amount of NCAM expressed. Interestingly, the percentage of membranous Ric8A in DRM is similar following NCAM180 overexpression in COS-7 cells (40%) and HEK293T cells (46%), or in NCAM expressing brain (44%), supporting the robustness of the observation in different systems. It is known that NCAM180 is present in both DRM, particularly lipid rafts, and DSM [32], [37], thus the interaction of NCAM180 and Ric8A can likely occur in DRM as well as in DSM. A possibility is that factors such as spectrin may interact with NCAM180 in DSM [31] to prevent or limit its interaction with Ric8A. Cooperative or antagonistic factors may control the localization of Ric8A to membrane subdomains NCAM180 being one of them. Indeed, if NCAM180 was the only factor, NCAM would be required for Ric8A localization in DRM and it would be absent from this compartment in NCAM knock out brains, which is not the case (Fig. 3D). Collectively, our findings lead us to propose that NCAM180 promotes the translocation of part of Ric8A to DRM, thereby amplifying β-adrenergic signaling locally in lipid rafts, thus resulting in an enhanced cAMP production. They open the way to investigations of other important events in the nervous sytem, such as long term potentiation in the dentate gyrus which requires NCAM [38] and depends on the activity of β-adrenergic receptors [39].

Interestingly, in neurons, which specifically express NCAM180, the proposed mechanism may intervene in neurotransmitter release for which NCAM180 and Ric8A are known to be required [11], [13], [14].

The extent to which the NCAM180-Ric8A interaction plays a role in other GPCRs signaling remains to be explored, as suggestions from other studies allow anticipating a more generalized role. Indeed, although the Ric8A null mutation is lethal, Ric8A heterozygous mice are viable and display some functional overlap with NCAM null mice, such as an anxious behavior. The anxious phenotype in NCAM knock out mice has been partly associated with an increased sensitivity to 5HT-1A agonist [16]. Moreover, expression of the NCAM180 in NCAM knock out mice was sufficient to rescue the behavioral and pharmacological phenotypes [17]. In summary, our data strengthen the fact that NCAM functions as a signaling molecule, but also demonstrate a novel role as a modulator of major signaling pathways in the cell. This is a previously unidentified mechanism of regulation of GPCR activity by a cell adhesion molecule, and may represent a potential therapeutic target.

## Supporting Information

Figure S1 **Specificity and efficacy of Ric8A knock down by small interfering RNA.** A- Typical image of COS-7 cells co-transfected with human Ric8A-GFP plasmid and siCtrl (a) or siRic8A (b), or co-transfected with pCXmb-Cherry plasmid and siCtrl (c) or siRic8A (d). Human Ric8A (green) was strongly reduced by siRic8A whereas Cherry (red) was not affected. Scale bar: a, b: 100 µm; c, d: 50 µm. B- Inhibition of endogenous Ric8A by siRic8A in HEK293T cells and HEK293T transfected with NCAM180 using α-tubulin constitutive protein as a reference. C- Quantitative analysis of Western blot illustrated in B using the ImageQuant software (Amersham Biosciences) as described previously (Marino *et al*, 2009).
(TIF)
