# Supplementary figures and images for: NCAM180 Regulates Ric8A Membrane Localization and Potentiates β-Adrenergic Response
Source: PLoS One. 2012 Feb 22;7(2):e32216. doi: 10.1371/journal.pone.0032216 (PMC3284568; doi:10.1371/journal.pone.0032216)

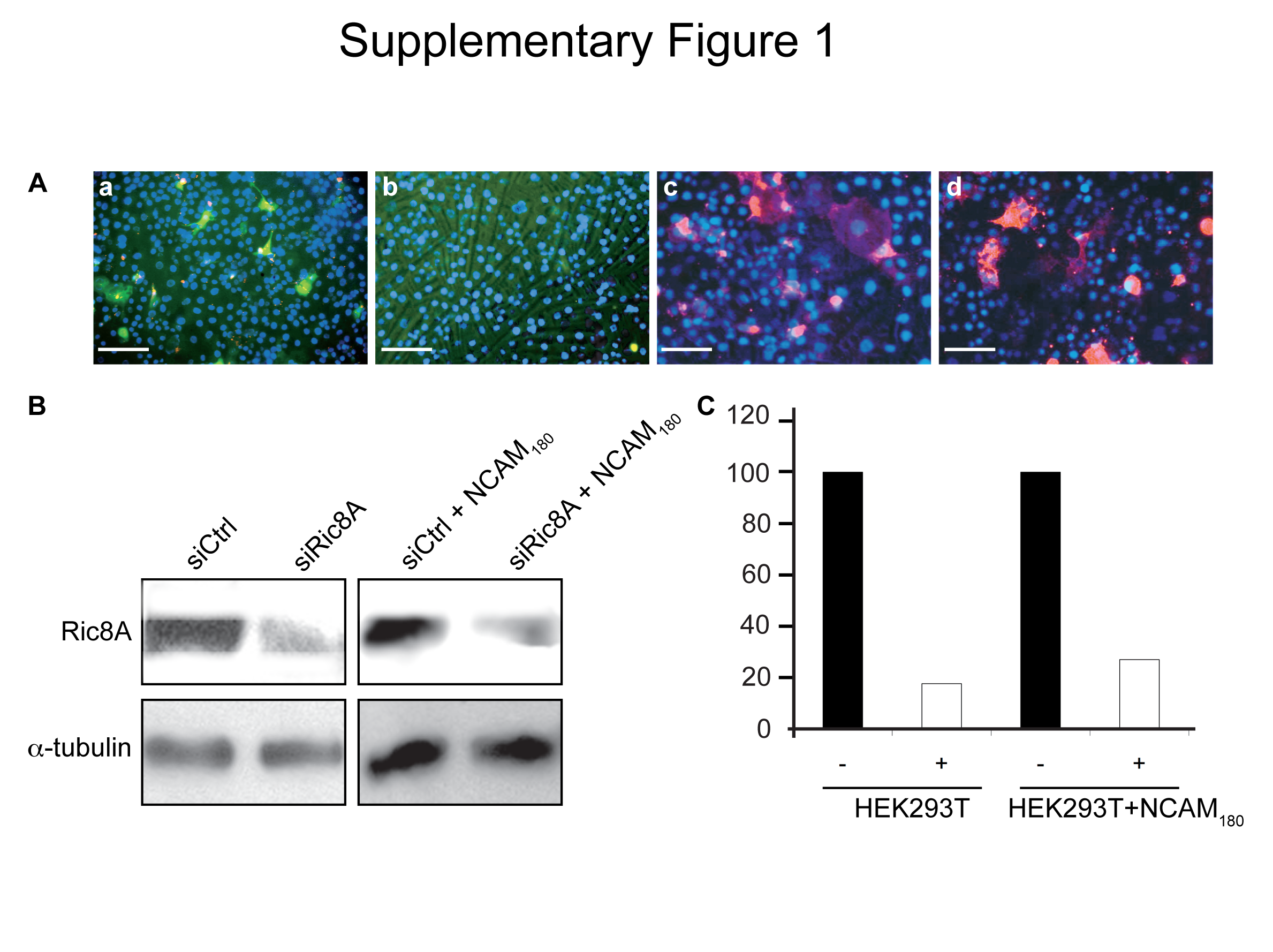

Supplement: Figure S1 — Specificity and efficacy of Ric8A knock down by small interfering RNA. A- Typical image of COS-7 cells co-transfected with human Ric8A-GFP plasmid and siCtrl (a) or siRic8A (b), or co-transfected with pCXmb-Cherry plasmid and siCtrl (c) or siRic8A (d). Human Ric8A (green) was strongly reduced by siRic8A whereas Cherry (red) was not affected. Scale bar: a, b: 100 µm; c, d: 50 µm. B- Inhibition of endogenous Ric8A by siRic8A in HEK293T cells and HEK293T transfected with NCAM180 using α-tubulin constitutive protein as a reference. C- Quantitative analysis of Western blot illustrated in B using the ImageQuant software (Amersham Biosciences) as described previously (Marino et al, 2009). (TIF) [file pone.0032216.s001.tif]
